# The Impact of Rubella Virus Infection on a Secondary Inflammatory Response in Polarized Human Macrophages

**DOI:** 10.3389/fimmu.2021.772595

**Published:** 2021-12-16

**Authors:** Erik Schilling, Anja Grahnert, Lukas Pfeiffer, Ulrike Koehl, Claudia Claus, Sunna Hauschildt

**Affiliations:** ^1^ Institute of Clinical Immunology, Medical Faculty, University of Leipzig, Leipzig, Germany; ^2^ Institute of Medical Microbiology and Virology, Medical Faculty, University of Leipzig, Leipzig, Germany; ^3^ Fraunhofer Institute for Cellular Therapeutics and Immunology, Leipzig, Germany; ^4^ Institute for Cellular Therapeutics, Hannover Medical School, Hannover, Germany; ^5^ Institute of Biology, University of Leipzig, Leipzig, Germany

**Keywords:** extracellular flux analysis, interferon, LPS, macrophage polarization, metabolism, TNF-α

## Abstract

Macrophages (MΦ) are known to exhibit distinct responses to viral and bacterial infection, but how they react when exposed to the pathogens in succession is less well understood. Accordingly, we determined the effect of a rubella virus (RV)-induced infection followed by an LPS-induced challenge on cytokine production, signal transduction and metabolic pathways in human GM (M1-like)- and M (M2-like)-MΦ. We found that infection of both subsets with RV resulted in a low TNF-α and a high interferon (IFN, type I and type III) release whereby M-MΦ produced far more IFNs than GM-MΦ. Thus, TNF-α production in contrast to IFN production is not a dominant feature of RV infection in these cells. Upon addition of LPS to RV-infected MΦ compared to the addition of LPS to the uninfected cells the TNF-α response only slightly increased, whereas the IFN-response of both subtypes was greatly enhanced. The subset specific cytokine expression pattern remained unchanged under these assay conditions. The priming effect of RV was also observed when replacing RV by IFN-β one putative priming stimulus induced by RV. Small amounts of IFN-β were sufficient for phosphorylation of Stat1 and to induce IFN-production in response to LPS. Analysis of signal transduction pathways activated by successive exposure of MΦ to RV and LPS revealed an increased phosphorylation of NFκB (M-MΦ), but different to uninfected MΦ a reduced phosphorylation of ERK1/2 (both subtypes). Furthermore, metabolic pathways were affected; the LPS-induced increase in glycolysis was dampened in both subtypes after RV infection. In conclusion, we show that RV infection and exogenously added IFN-β can prime MΦ to produce high amounts of IFNs in response to LPS and that changes in glycolysis and signal transduction are associated with the priming effect. These findings will help to understand to what extent MΦ defense to viral infection is modulated by a following exposure to a bacterial infection.

## Introduction

Macrophages (MΦ) are a vital part of the innate immune response. They comprise different activation states that have been generally categorized into two broad but distinct subsets termed M1-MΦ (classically activated) and M2-MΦ (alternatively activated). While M1-MΦ triggered by interferon (IFN)-γ in combination with Toll-like receptor (TLR) agonist lipopolysaccharide (LPS) promote pro-inflammatory and tumoricidal properties, M2-MΦ generated in response to IL-4 and IL-13 show anti-inflammatory and pro-tumoral properties and serve key roles in wound healing and tissue repair. These classes are generally accepted to be at the opposite extremes of a spectrum of intermediate phenotypes (1).

However, next to the generation of M1 and M2 phenotypes other models use growth-factors namely GM- and M-CSF to generate pro- and anti-inflammatory MΦ, respectively. These factors reflect *in vivo* conditions as M-CSF has been implicated in the steady state control of macrophage development whereas levels of GM-CSF are elevated during inflammatory reactions. As MΦ developed in the presence of CSFs do not exactly mirror those of the activated M1- and M2-MΦ we will refer to the two subsets used here as GM- and M-MΦ, respectively (2).

Besides tissue development, homeostasis and tissue repair MΦ play a crucial role in immunity to pathogens including both bacteria and viruses. They respond to these pathogens by producing a variety of mediators including cytokines like TNF-α and IFNs. When exposed to LPS TNF-α is mainly released by pro-inflammatory MΦ, while anti-inflammatory MΦ only produce low levels of this cytokine, but secrete instead high amounts of IFNs (2).

In contrast to the well-defined LPS-induced cytokine response of the two MΦ subsets, the role of the different phenotypes in mediating TNF-α and IFN release upon viral infection has been less extensively studied.

As reviewed lately by Nikitina et al., monocytes and MΦ, cells with an extended life span and access to tissues, can be subverted by multiple viruses from different families (3). These viruses include RNA viruses such as Chikungunya virus, which persists in MΦ (4), as well as DNA viruses such as human cytomegalovirus, which was shown to replicate in MΦ up to 16 weeks after infection (5). The reproductive cycle of retroviruses including human immunodeficiency virus (HIV) involves latency and chronic replication in monocytes and MΦ as reviewed by Le Douce et al. (6). Several lines of evidence support the relevance of MΦ for the pathogenesis of the single-stranded, plus-sense RNA virus rubella virus (RV). RV is transmitted *via* aerosols and after initial replication in the respiratory tract spreads to local lymphnodes. This leads to lymphadenopathy as one of the early detectable symptoms of rubella infection (7). It is very likely that MΦ serve as targets of RV within the lymphnodes and that, as suggested by van der Logt et al. (8), the virus replicates within these cells. Additionally, viral antigen was detected in human MΦ in clinical samples. The antigen was found in alveolar MΦ in tissue samples of children born with fatal congenital rubella (9) and in M2-MΦ present in RV-associated cutaneous granulomas of patients with primary immunodeficiency disorders (PID) (10).

It is especially noteworthy, that RV can replicate in both human MΦ types (11) thus allowing a direct comparison of virus infection-associated changes in the two subsets.

In view of the still ill-defined role of human MΦ in RV dissemination in the body and their impact on the associated pathogenesis, we here asked to what extend RV affects immune functions and metabolic profile of MΦ and how RV alters their response when exposed to LPS as a second stimulus. This experimental approach will deepen our understanding into the competence of MΦ to respond to RV and into RV-induced reactions, that become apparent after a coinfection.

We found that infection of GM- and M-MΦ with RV alone or followed by a challenge with LPS alters their cytokine release, metabolic activity and signal transduction pathways. The here presented data underline the relevance of dissecting the cellular responses to the coinfection to better understand the RV infection-induced effects on the innate immune system.

## Material and Methods

Unless otherwise indicated, materials used in this study were from the following manufacturers: Sigma-Aldrich (Taufkirchen, Germany): fetal calf serum (FCS), LPS from *E. coli* (serotype 055:B5); Seromed Biochrom KG (Berlin, Germany): penicillin, streptomycin; GE Healthcare (Little Chalfont, Buckinghamshire, UK): RPMI 1640 (with L-glutamine, 25 mM HEPES and phenol red), Ficoll-Paque™ Plus.

Recombinant human IFN-β was purchased from Peprotech (Hamburg, Germany) and the monoclonal antibody against RV capsid (C) protein (clon 2-36) from Meridian Life Science, Inc. (Memphis, TN, USA). Material and reagents for measuring cellular metabolism by extracellular flux analysis with the Seahorse technology were purchased from Agilent Seahorse Technologies (Santa Clara, CA, USA).

### Cell Separation and Cell Culture

Buffy coats of healthy donors were acquired from the blood service (Institute of Transfusion Medicine University Hospital Leipzig; ethics license 272-12-13082012). Human peripheral blood mononuclear cells were obtained from buffy coats by Ficoll-Paque Plus density centrifugation. After washing with PBS containing 0.3 mM EDTA, monocytes were isolated by counter-flow elutriation using the JE-5.0 elutriation system (Beckman Coulter, Brea, CA, USA), as described previously (12).

Monocytes (5×10^5^/ml) with a purity of at least 90%, as assessed by flow cytometry using anti-CD14-APC Ab (M5E2, BioLegend, San Diego, CA, USA) were suspended in RPMI 1640 medium supplemented with 10% (v/v) FCS, 100 U/ml penicillin and 100 mg/ml streptomycin and differentiated with 500 IU/ml GM-CSF (Leukine, sargramostim) to GM-MΦ or 50 ng/ml M-CSF (R&D Systems, Minneapolis, MO, USA) to M-MΦ at 37°C and 5% CO_2_ in teflon bags (Zell-Kontakt, Nörte-Hardenberg, Germany; fluorinated ethylene propylene foil, 50 µm, hydrophobic). After seven days, MΦ (5×10^5^/ml) were suspended in RPMI 1640 medium supplemented with 10% (v/v) FCS, 100 U/ml penicillin and 100 mg/ml streptomycin and incubated for 2 h in cell culture plates before starting experiments. Flow cytometry analysis of MΦ cell surface markers was performed as described previously (13), using direct dye-labelled antibodies anti-CD14-APC (M5E2, BioLegend), anti-CD40-PerCp (Elabscience Biotechnology, Houston, TX, USA), anti-CD80-PE (L307, BD Pharmingen, San Jose, CA, USA) and the unlabelled antibody anti-CD163 (GHI/61 BD Pharmingen) in combination with a FITC-labelled goat anti-mouse secondary antibody.

### Virus Infections

Virus infection was carried out as previously described (11). Stock virus preparations of the low-passaged (up to passage 10) clinical isolate RVi/Wuerzburg.DEU/47.11_12-00009 (Wb-12) were prepared and titered by standard plaque assay on Vero cells (green monkey kidney epithelial cell line, ATCC CCL-81). Vero cells were cultured in Dulbecco’s modified Eagle’s medium (DMEM; Thermo Fisher Scientific) with 10% (v/v) FCS. Human MΦ were infected with RV (approved by the ethics committee at the Medical Faculty of the University of Leipzig; ethics license 001/19-ek) at a multiplicity of infection (MOI) of 1.5. Cells that were not infected (mock) or treated with RV inactivated by exposure to UV-light (750,000 μJ/cm^2^ (UV-Stratalinker 2000, Stratagene)) (UV-RV) served as controls. UV-RV was applied at the same MOI as RV. Before infection culture media were discarded and RV or the respective controls (mock and UV-RV) were added. After 2 h of incubation the media were removed, MΦ were washed with PBS and suspended in fresh culture media.

### Immunofluorescence Microscopy

The immunofluorescence analysis of RV-infected MΦ was performed as described previously (14). Briefly, MΦ were cultivated on glass slides, washed at 24 hours post-infection (hpi) with PBS, and fixed with 2% (w/v) paraformaldehyde (PFA) in PBS for 15 min. After fixation, cells were washed with PBS and permeabilized with 0.3% Triton X-100 (v/v) in PBS at 37°C for 30 min followed by blocking with 0.3% Triton X-100 (v/v) and 5% normal goat serum (v/v) in PBS for 30 min at 37°C in a humidified chamber. Thereafter cells were incubated with primary mouse monoclonal antibody to rubella capsid protein (clone 2-36; 1:200 dilution; Meridian Life Science Inc, Saco, ME USA) for 60 min at 37°C followed by a three-time washing step with PBS. After incubation with the Cy3 conjugated donkey anti-mouse IgG secondary antibody (1:200 dilution; Dianova, Hamburg, Germany) for 45 min, the cells were washed again three times with PBS. To label F-actin, a 1:40 dilution of Alexa Fluor 488 phalloidin (Thermo Fisher Scientific) was added followed by DNA counterstaining during the mounting step with Fluoromount G (Invitrogen Life Technologies, Thermo Fisher Scientific, Schwerte, Germany*)* containing DAPI at a suitable working concentration. Stained cell samples were analyzed with a Leica TCS SP8 laser scanning confocal microscope and processed using Corel DRAW x7 with slight alterations to brightness and contrast.

### Detection of Intracellular Virus Genome Copies

Viral genome copies were determined after isolation of total cellular RNA by ReliaPrep™ RNA Miniprep System (Promega) followed by one-step Taq Man RT-qPCR as described previously (15).

### Measurement of TNF-α and IFNs in Culture Supernatants

TNF-α and IFNs were determined in culture supernatants of MΦ (5×10^5^/ml) using a human TNF-α ELISA (PeproTech) or a LEGENDPLEX human type 1/2/3 Interferon panel (5-plex) kit (BioLegend), respectively according to the manufacturer’s protocol.

### Western Blot Analysis

Western blot analysis was carried out as described previously (12). After washing with PBS, MΦ (5 ×10^5^) on culture plates were suspended in 100 µl RIPA buffer (50 mM Tris, 150 mM NaCl, 1% Nonidet P 40, 0.5% deoxycholate, 0.1% SDS; pH 7.5) supplemented with Complete EDTA-free protease inhibitor cocktail (Roche Diagnostics, Mannheim, Germany) and with phosphatase inhibitors (1 mM Na_3_VO_4_ and 50 mM NaF). After sonication on ice, samples were centrifuged for 5 min at 15,000 g and 4°C. In resulting supernatants protein concentrations were determined using a DC Protein Assay (Bio-Rad, Hercules, CA, USA) according to the manufacturer’s protocol. Cell lysates (25–30 µg) were boiled in 1× Laemmli sample buffer, run on a 12% SDS-polyacrylamide gel (Protean II, Bio-Rad GmbH) and transferred to polyvinylidene difluoride membranes (PVDF membrane, Amersham Biosciences, Munich, Germany).

After blocking with 5% milk powder, PVDF membranes were probed with the following antibodies: anti-phospho-Stat-1 rabbit Ab (Tyr701, D4A7, 1: 1,000); anti-phospho-NF-κB rabbit Ab (p65, Ser536, 93H1, 1: 1,000); anti-phospho-TBK1 rabbit Ab (Ser172, D52C2 XP^®^, 1: 1,000); anti-phospho-ERK1/2 mouse Ab (Thr202/Tyr204, E10, 1: 2,000); anti-phospho-c-Jun amino-terminal kinase (JNK) rabbit Ab (Thr183/Tyr185, 81E11, 1: 1,000) (all from Cell Signaling, Danvers, MA, USA), anti-rubella (E1) mouse Ab (1: 500, Merck, Darmstadt, Germany) or anti-β-actin mouse Ab (clone AC 74, 1: 2,000, Sigma-Aldrich, St. Louis, USA). Primary Antibody were detected with the following POD-conjugated secondary antibodies: goat anti-rabbit IgG Ab (1: 20,000, Dianova) or goat anti-mouse IgG Ab (1: 8,000, Sigma-Aldrich, St. Louis, USA). Chemiluminescent detection on membranes by using ECL-A/ECL-B substrate (both from Sigma Aldrich) or SuperSignal West Femto Maximum Sensitivity Substrate (Thermo Fisher Scientific) were analyzed on a CCD-camera Stella (raytest Isotopenmessgeräte GmbH, Straubenhardt, Germany). For Densitometric analysis, the AIDA Image Analyzer Software (Elysia-raytest GmbH, Straubenhardt, Germany) was used and sample values were expressed as density relative to β-actin. For stripping the membrane, blots were washed 5 min with distilled water followed by a 3 times incubation with 0.1 M glycine-HCl (pH 2.0) for 5 min. After an additional washing step, membranes were again blocked with 5% milk powder before re-probing with the next primary antibody.

### RNA Isolation and Reverse Transcription

Macrophages (3×10^5^) were washed with PBS and total RNA was isolated using the RNeasy Mini Kit (Qiagen, Hilden, Germany), according to the manufacturer’s protocol. DNase I treatment and reverse transcription of equal amounts of RNA (at least 250 ng) to cDNA were performed as previously described (12).

### Real-Time PCR (qPCR)

For real-time PCR 1.5 µl of cDNA template were added to a reaction mixture containing 7.5 µl of the SYBR Green PCR mastermix (Bio-Rad, Hercules, CA, USA), 250 nM forward and reverse primers (see below) in a final volume of 15 µl. The following primers were used: GNB2L1 (fwd 5`-GAGTGTGGCCTTCTCCTCTG-3`; rev 5`-GCTTGCAGTTA GCCAGGTTC-3`) (16), IFN-β (fwd 5`-AACTTTGACATCCC TGAGGAGATTAAGCAG-3`; rev 5`-GACTATGGTCCAGGC ACAGTGACTGTACTC-3`) (17), IFN-λ1 (fwd 5`-GCAGGTTC AAATCTCTGTCACC-3`; rev 5`-AAGACAGGAGAGCTGC AACTC-3`) (18), IFNAR1 (fwd 5`-TCAGGTGTAGAAGAAA GGATTGAAA-3`; rev 5`-AGACACCAATTTTCCATGACGT A-3`) (19), IFNLR1 (fwd 5`-TGGGTGGAGTCCGAATACCT-3`; rev 5`-GAGTGATCTGGACTGGCTGG-3`).

IFNLR1 primers were designed using the BLAST and the Primer3 program. The PCR reactions were performed using the CFX connect real-time PCR detection system (Bio-Rad, Hercules, CA, USA) according to the following protocol: initial denaturation at 95°C for 10 min, followed by 40 cycles at 95°C for 15 s (denaturation), at 60°C for 30 s (primer annealing) and at 72°C for 30 s (extension/synthesis). Product quantification was carried out at 72°C. Negative controls using water as template were run under the same conditions. Gene expression was calculated using the ΔΔCt method as previously described with *GNB2L1* (Guanine nucleotide-binding protein subunit beta-2-like 1) as the reference gene (20).

### Metabolic Assessment Through Extracellular Flux Analysis: Analysis of the Oxygen Consumption Rate (OCR) and the Extracellular Acidification Rate (ECAR)

The OCR and ECAR were measured using an XFp analyzer (Agilent Seahorse Technologies, Santa Clara, CA, USA). XFp Seahorse plates were seeded with MΦ (200 µl per well) at a density of 2x10^5^/ml. After 2 h cells were analyzed or subjected to virus infection. Thereafter culture medium was replaced by XF base medium (Agilent Seahorse Technologies) supplemented with 2 mM glutamine and 11 mM glucose. The cells were then incubated at 37°C in a CO_2_-free incubator for 45 to 60 min. Hereafter basal OCR and ECAR were determined in the XFp analyzer, before LPS (100 ng/ml) was injected (measurement point 6). Further measurements were taken for another 1 h. Agilent Seahorse software Wave 2.3 was used for data analysis.

### Statistical Analysis

Statistical analysis was done using GraphPad PRISM or SigmaPlot^®^ software. Statistical significance was calculated with Student’s *t*-test or ANOVA test as indicated in the respective figure legend. Statistical significance is classified as follows: * p ≤ 0.05, ** p ≤ 0.01, *** p ≤ 0.001. ANOVA test of percentage or fold change data was performed after log transformation.

## Results

### RV Infection and Cytokine Response

**Figure 1:**
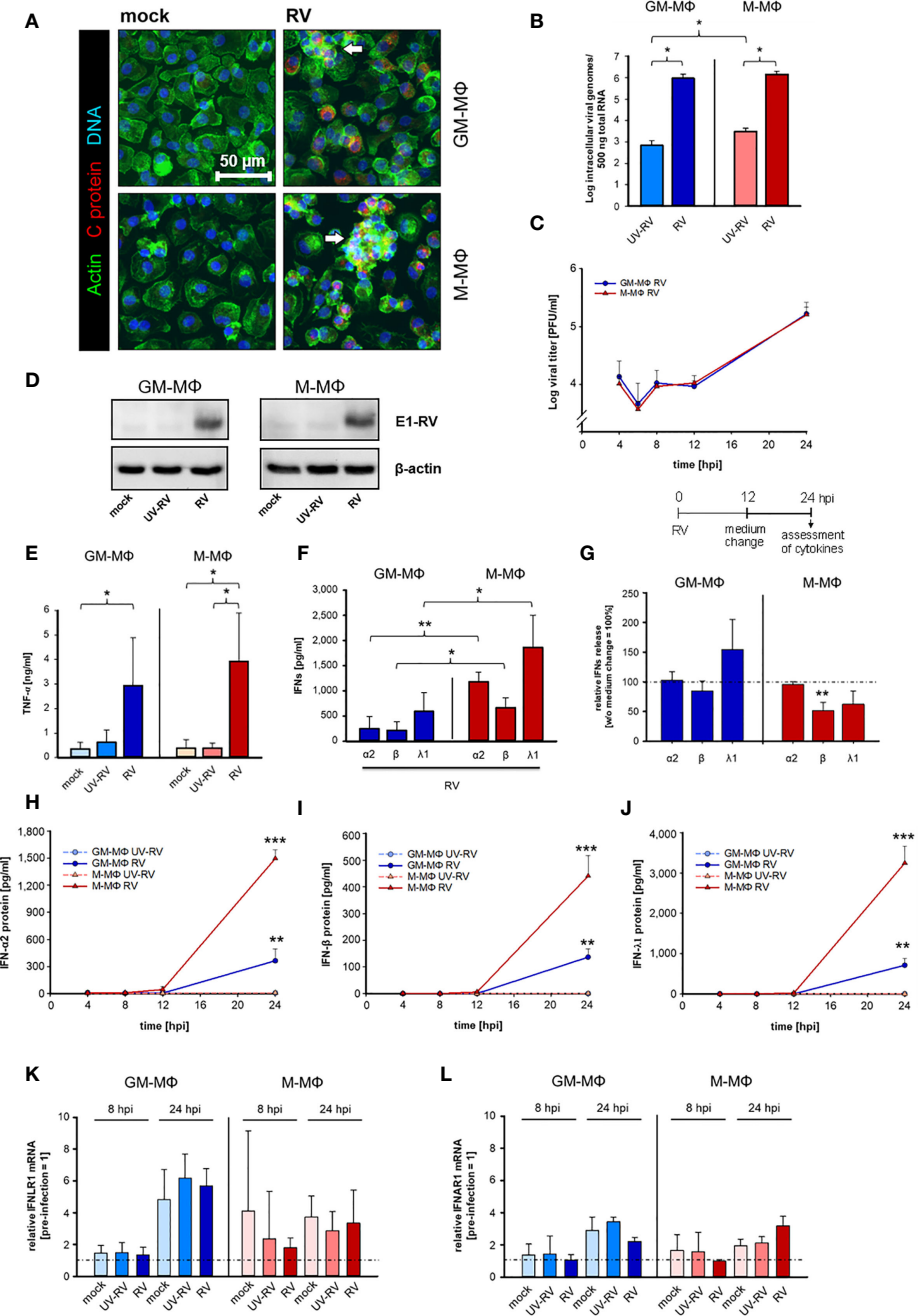
Kinetics of RV replication and RV-induced cytokine response in human MΦs. **(A)** Immunofluorescence staining of capsid protein (C protein, red), actin (green) and DNA (blue) of RV-infected MΦ and mock controls, 24 hours post infection (hpi). Arrows mark groups of cells arranged in clusters (n = 3). **(B)** GM-MΦ and M-MΦ (5x10^5^/ml) were infected with RV or ultraviolet light-inactivated RV (RV-UV) and the number of intracellular viral genome copies was determined by quantitative one-step TaqMan PCR at 24 hpi. Data represent means ± SD (n = 3). Statistical analysis was performed using the ANOVA test. **(C)** Assessment of extracellular virus particles by plaque assays of supernatants of GM-MΦ and M-MΦ collected at indicated time points after infection (n = 3 to 5). Data represent means ± SD. **(D)** Cells (5x10^5^/ml, 24 hpi) were lysed using RIPA buffer and proteins separated by SDS-PAGE were subjected to western blot analysis using antibodies for the rubella E1 structure protein and β-actin. One representative blot out of three is shown. At 24 hpi **(E)** secreted TNF-α was determined by ELISA (n = 6, means ± SD) and **(F)** IFN protein levels by LEGENDPLEX human interferon panel kit (n = 3, means ± SD). Statistical analysis for **(E, F)** were performed using the ANOVA test. **(G)** At 12 hpi the medium was changed and the cells were incubated for another 12 h. At 24 hpi the amount of IFNs produced after a medium change (12 hpi) was calculated as fold induction without medium change (= 100%). Data represent means ± SD (n = 3). Statistical analysis was performed using the ANOVA test. At indicated time points the protein expression levels of IFN-α2 **(H)**, IFN-β **(I)** and IFN-λ1 **(J)** were quantified by LEGENDPLEX human interferon panel kit. Data represent means ± SD (n = 3). Student’s *t*-test and significances were calculated to MΦ treated with RV-UV. IFNLR1 **(K)** and IFNAR1 **(L)** mRNA expression were determined by qPCR before RV infection as well as 8 and 24 hpi. Data are presented as relative mRNA expression of pre-infected MΦ (= 1) (n = 3, means ± SD) *p ≤ 0.05, **p ≤ 0.01, ***p ≤ 0.001.

Before infecting cells we verified the well documented phenotypical characteristics of GM- and M-MΦ (21) by showing that M-MΦ specifically expressed CD163 (M-MΦ 613.5 ± 663.6; GM-MΦ 23.5 ± 15.2) and CD14 (M-MΦ 5094.0 ± 773.7; GM-MΦ 1700.3 ± 933.1), while expression levels of CD80 (GM-MΦ 31.7 ± 8.4; M-MΦ 7.0 ± 7.2) and CD40 (GM-MΦ 167.7 ± 29.3; M-MΦ 65.7 ± 17.0) were constitutively higher on GM- than on M-MΦ. Based on recent findings, showing that after RV-infection neither the amount of extracellular virus particles nor the number of RV-positive cells varied between GM- and M-MΦ (11), we here examined the cellular morphology of both subsets after infection with RV by staining of F-actin. We found cluster formation (**Figure 1A**) which was slightly more prevalent in M-MΦ than in GM-MΦ.

Similar to RV, UV-inactivated RV (UV-RV) was detected in both cell types. However, the genome copies, which hardly varied between GM- and M-MΦ were 2 to 3 log steps lower than after RV infection indicating that UV-RV does not replicate within the cell and that the copies reflect the original incoming virus genome (**Figure 1B**).

The fact that UV-RV is taken up by human MΦ without initiation of RV replication and that UV-RV contains the virion components as well as factors released from Vero cells during virus stock preparation, make it a useful control when studying RV infection.

According to the time course of virion production performed over 24 h the number of infectious virus particles generated by GM- and M-MΦ was almost identical between 4 and 24 h post infection (hpi) (**Figure 1C**). At 6 hpi the number slightly decreased (indicating the eclipse phase) and at 24 hpi after a delay phase between 8 and 12 hpi virion production and release to the medium were increased.

The similarity of RV replication rates between GM-MΦ and M-MΦ was also reflected by expression levels of viral E1 protein as detected by western blot analysis at 24 hpi (**Figure 1D**). Thus, possible differences between GM- and M-MΦ in LPS-induced responses are unlikely to be due to differences in the course of infection.

Next, we determined the impact of RV infection on the inflammatory response of GM- and M-MΦ by analyzing the production of the cytokines TNF-α, IFN-α2 and IFN-β (type I), IFN-γ (type II), IFN-λ1 and λ2/3 (type III). IFN-γ and IFN-λ2/3 were not detectable under any assay conditions.

As seen in **Figure 1E**, RV induces TNF-α production in both subtypes, the amounts secreted by M-MΦ slightly exceeding those of GM-MΦ.

GM- and M-Mϕ also responded to the RV-infection with an increase in IFN-α2, IFN-β and IFN-λ1 concentrations (**Figure 1F**), whereas no IFN-γ was detectable (data not shown).

The observation that the increase of virion production (**Figure 1C**) also started 12 hpi and that a medium change carried out 12 hpi had no inhibitory effect on RV-induced IFN production (except IFN-β) (**Figure 1G**) points to a close relation between virion generation and IFN production. M-MΦ produced far more IFNs than GM-MΦ, the concentrations of IFN-λ1 being highest in both subtypes. UV-RV and mock controls did not produce any IFNs.

These differences in IFN-α2, -β and -λ1 production can hardly be explained by the kinetics of their release. Secretion of all three IFNs started at 12 hpi and high concentrations were reached at 24 hpi (**Figures 1H–J**). 

As the expression levels of IFN-receptors play an important role for the production of IFNs in a positive feedback loop we determined the mRNA receptor levels of type III interferon-lambda receptor 1 (IFNLR1) and type I interferon-α/β receptor 1 (IFNAR1) before infection with RV as well as at 8 and 24 hpi. Before starting the experiments, we addressed the expression level of both types of receptors and showed that they hardly differed between uninfected GM- and M-MΦ (relative to M-MΦ expression = 1, GM-MΦ IFNAR = 0.95 ± 0.14, IFNLR1 = 1.24 ± 0.16). At 24 hpi IFNLR1 transcripts increased in GM- and M-MΦ about 5- and 3-fold respectively as compared to 0 h controls (before treatment) (**Figure 1K**) and at 8 hpi only a minor increase was observed in M-MΦ.

We also found IFNAR1 transcript to be moderately elevated in both subtypes at 24 hpi; the small rise at 8 hpi was restricted to mock- and UV-RV-infected controls (**Figure 1L**). However, as the RV-induced rise of both IFN receptor transcripts in GM- and M-MΦ also occurred in mock and UV-RV, a correlation between increased mRNA levels of the receptors and an elevated RV-induced IFN-response cannot be drawn.

These data show that RV infects GM- and M-MΦ to a similar extent and that both subtypes are competent to respond to RV mounting a cytokine response consisting of a release of a relative minor elevated TNF-α production and an increase in type I IFN-concentrations, the latter being more pronounced in M-MΦ than in GM-MΦ.

### RV Supports IFN-Production After a Secondary Exposure to LPS

Having shown that RV induces TNF-α and IFN-production in both MΦ subtypes we next tested whether stimulation with LPS after RV infection affects the outcome of the cytokine response.

**Figure 2:**
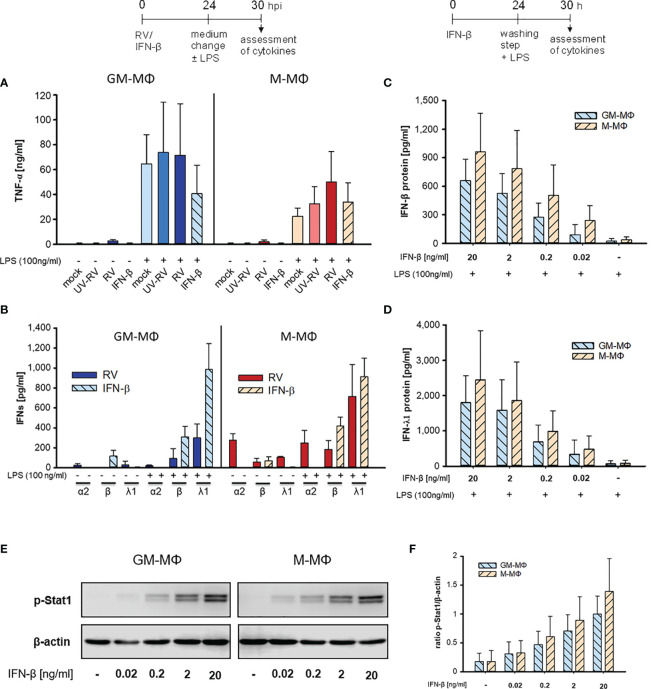
LPS-induced cytokine response after infection with RV and IFN-β. At 24 h after incubation of mock, UV-RV, RV and IFN-β (20 ng/ml) treated MΦ (5x10^5^/ml) the medium was changed and the cells were incubated in the presence and absence of LPS (100 ng/ml). After 6 h TNF-α **(A)** and IFN-concentrations **(B)** in the culture supernatants were determined by ELISA and LEGENDPLEX human interferon panel kit, respectively. Data represent means ± SD (n = 3). **(C, D)** GM-MΦ and M-MΦ (5x10^5^/ml) were incubated with decreasing concentrations of IFN-β (20; 2; 0.2; 0.02 ng/ml) for 24 h. After washing with PBS cells were stimulated with LPS (100 ng/ml) for 6 h. IFN-concentrations were determined by LEGENDPLEX human interferon panel kit. Data represent means ± SD (n = 3). **(E, F)** GM-MΦ and M-MΦ (5x10^5^/ml) were incubated with increasing concentrations of IFN-β (0.02; 0.2; 2; 20 ng/ml). After 24 h cells were lysed using RIPA buffer and proteins separated by SDS-PAGE were subjected to western blot analysis using antibodies for p-Stat1 and β-actin. One representative blot out of three is shown **(E)**. **(F)** Western blot bands were quantified and p-Stat1 normalized to loading control β-actin. Data represent means ± SD (n = 3).

Therefore, cells were infected with RV and the respective controls (mock, UV-RV) for 24 h before fresh medium with or without LPS was added for another 6 h. As seen in **Figure 2A**, GM- and M-MΦ infected with RV only, produced small amounts of TNF-α (see **Table 1**, **Figure 2A**). The TNF-α levels increased after exposure to LPS, the rise being largely independent of the viral infection, albeit a small but insignificant increase was observed in M-MΦ after RV infection (**Figure 2A**).

**Table 1:** LPS-induced cytokine response of RV-infected MΦ.

GM-MΦ				M-MΦ			
TNF-α (ng/ml)	IFN-α2 (pg/ml)	IFN-β (pg/ml)	IFN-λ1 (pg/ml)	TNF-α (ng/ml)	IFN-α2 (pg/ml)	IFN-β (pg/ml)	IFN-λ1 (pg/ml)
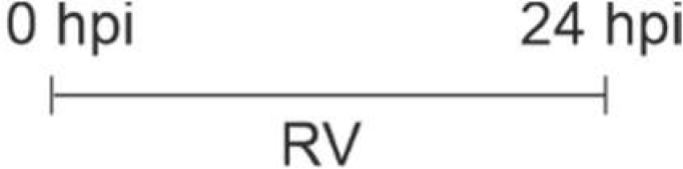							
3 ± 2	251 ± 246	219 ± 170	594 ± 376	4 ± 2	1182 ± 191	666 ± 199	1866 ± 637
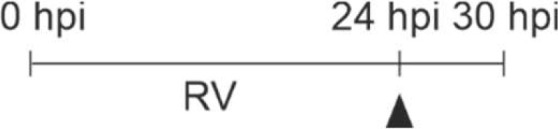							
3 ± 1	27 ± 15	n.d.	28 ± 39	2 ± 2	276 ± 64	58 ± 35	108 ± 7
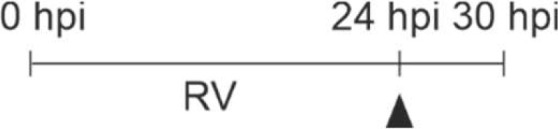							
72 ± 42	21 ± 8	94 ± 98	300 ± 140	50 ± 25	247 ± 129	183 ± 90	714 ± 323
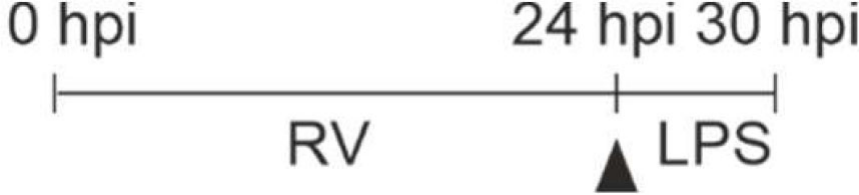							
65 ± 24	n.d.	n.d.	n.d.	23 ± 7	n.d.	n.d.	n.d.

RV-infected GM- and M-MΦ (5x10^5^/ml) and mock controls were either incubated for 24 h or 24 h followed by a 6 h incubation in the presence or absence of LPS (100 ng/ml). TNF-α and IFNs concentrations were determined in culture supernatants by ELISA and LEGENDPLEX human interferon panel kit, respectively (n = 3; means ± SD). The experimental set-up is visualized by a graphical scheme. Arrow heads indicate medium change with or without addition of LPS. The last time point in the graphical scheme refers to the time point of analysis. n.d., Not detectable.

Both subtypes secreted small amounts of IFNs (except for IFN-β in GM-MΦ) after RV infection in the absence of LPS (**Figure 2B**, **Table 1**). However, addition of LPS to the infected cells resulted in an increase of IFN-β and IFN-λ1, but not of IFN-α2 levels. Neither UV-RV infected (data not shown) nor mock controls (**Table 1**) produced any IFNs in response to LPS.

As IFN-β, known to act in an autocrine fashion (22), was produced by GM- and M-MΦ in response to RV-infection (**Figure 1F**), we tested whether it can mimic the effects of RV on LPS-induced cytokine production when added exogenously. We found that IFN-β (20 ng/ml) did not induce TNF-α production whereas after challenge with LPS the TNF-α levels increased, in M-MΦ similar to controls and in GM-MΦ even less (**Figure 2A**). Treatment with IFN-β also failed to induce IFN production (**Figure 2B**). Only after exposure to LPS IFN-β and IFN-λ1 levels increased, IFN-α2 levels were unaffected. The small amounts of IFN-β present in the absence of LPS likely stem from the added IFN-β (**Figure 2B**).

When diluting IFN-β concentrations starting with 20 ng/ml the LPS-induced enhancement of IFN-β and IFN-λ1 production decreased in a concentration dependent manner (**Figures 2C, D**). As little as 0.2 to 0.02 ng/ml were sufficient to amplify the IFN-β and -λ1 response. M-MΦ produced slightly more IFN-β and -λ1 than GM-Mϕ and the IFN-λ1 release always exceeded that of IFN-β.

As seen in **Figures 2E, F** exposure of GM- and M-MΦ to small concentrations (0.2 to 0.02 ng/ml) of IFN-β results in low phosphorylation levels of Stat1, sufficient for enhanced responsiveness to the secondary stimulus LPS (**Figures 2C, D**).

Taken together our data show that RV-infection hardly effects the LPS-induced TNF-α production and that the virus primes both subtypes to produce appropriate amounts of IFN-β and -λ1 in response to LPS. The same holds true at least for IFN-β and -λ1 production when RV is replaced by IFN-β.

**Figure 3:**
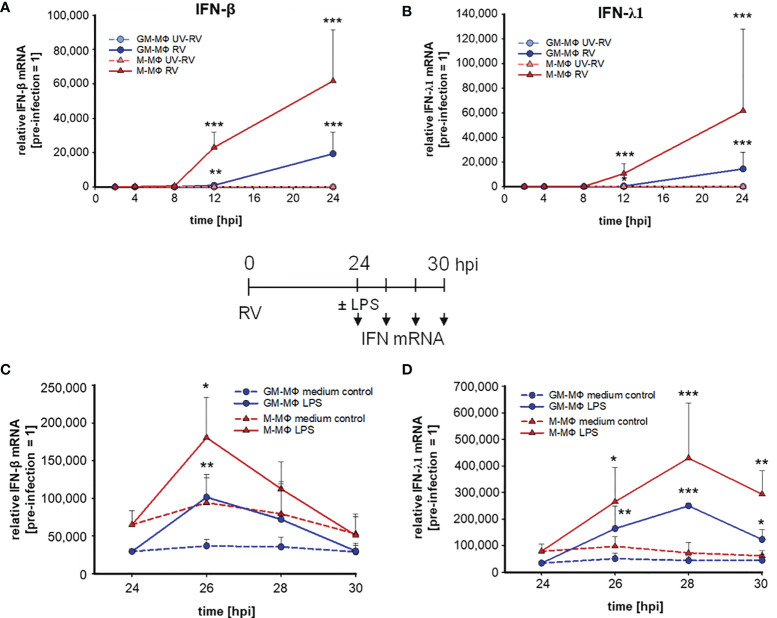
LPS-induced IFN mRNA expression after infection with RV. RV and UV-RV-infected MΦ (5x10^5^/ml) were incubated for 2, 4, 8, 12 and 24 h. IFN-β **(A)** and IFN-λ1 **(B)** mRNA levels were quantified by qPCR relative to pre-treated MΦ (0 h =1). Data of RV-infected cells are shown and represent means ± SD (n = 3). Statistical analysis was performed using the ANOVA test and significances were calculated to MΦ infected with RV-UV. At 24 hpi RV-infected cells were treated with medium (control, dashed line) or incubated with LPS (100 ng/ml) and subjected to RNA extraction after 2, 4 and 6 h of incubation. IFN-β **(C)** and IFN-λ1 **(D)** mRNA levels were quantified by qPCR relative to pre-infected MΦ (0 h =1). Data represent means ± SD (n = 3). Statistical analysis was performed using the ANOVA test and significances were calculated to medium control. *p ≤ 0.05, **p ≤ 0.01, ***p ≤ 0.001.

Next, we tested whether the priming effect of RV on IFN-β and -λ1 production also occurred at the mRNA level. The mRNA expression of IFN-β (**Figure 3A**) and -λ1 (**Figure 3B**) started to increase at 8 hpi and 12 hpi in M- and GM-MΦ respectively and continued to rise up to 24 hpi. At all time points the values reached by M-MΦ exceeded those of GM-MΦ (**Figures 3A, B**). Negligible amounts of IFN-mRNA were produced after UV-RV infection. Extending the incubation time for another 6 h after medium change, in the presence of LPS resulted in a further increase of IFN-β (**Figure 3C**) and -λ1 (**Figure 3D**) mRNA in both MΦ subtypes.

The mRNA level of IFN-β in LPS challenged GM- and M-MΦ peaked after 2 h and reached basal values after 6 h. IFN-λ1 mRNA expression, however, peaked after 4 h in both subtypes and declined thereafter. In the absence of LPS the production of IFN-β and -λ1 mRNA remained at a rather constant level (**Figures 3C, D**).

Thus, IFN-β and -λ1 protein kinetics up to 24 hpi (**Figures 1I, J**) clearly reflect the corresponding mRNA expression levels (**Figures 3A, B**) and the IFN-β and -λ1 mRNA levels as well as the protein concentrations reached by M-MΦ 6 h after exposure to LPS both exceeded those of GM-MΦ (**Table 1**).

Taken together these data demonstrate that RV as well as IFN-β regulate the IFN-production in response to a secondary challenge with LPS. Under assay conditions that result in a very low LPS-induced IFN-production the virus primes both macrophage subsets to react to LPS treatment with a rise in IFN concentrations. The same holds true when substituting RV by IFN-β. Notably low concentrations of IFN-β (pg/ml) are sufficient for enhanced LPS-induced responsiveness to this cytokine.

### RV Impairs LPS-Induced ERK1/2 Activation

**Figure 4:**
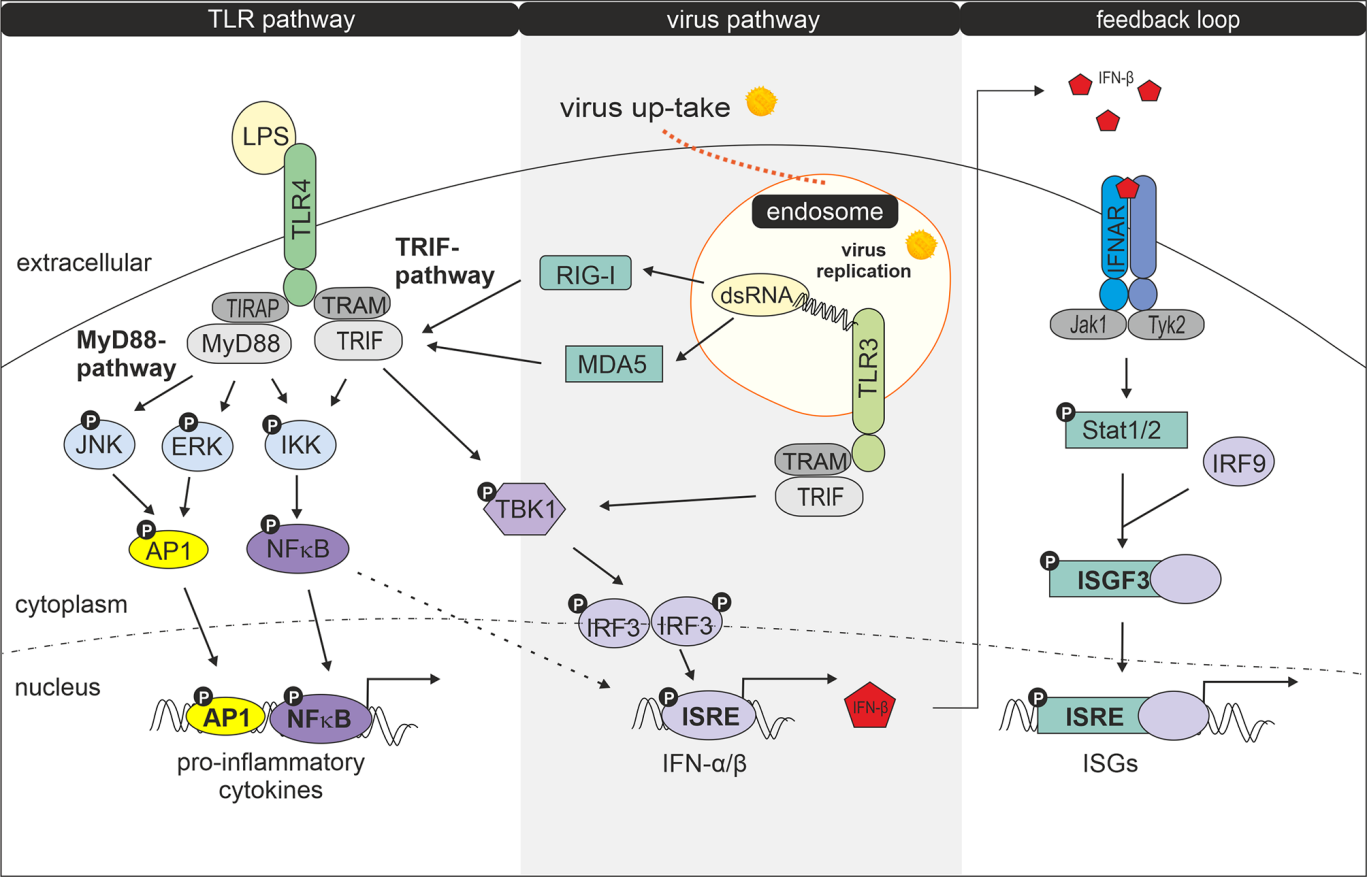
Interaction of LPS- and virus-induced signalling pathways. Modified according to Randall et al. (23). Following uptake and replication of RV double stranded RNA (dsRNA) is generated. Recognition of dsRNA by cytosolic receptors MDA5 (melanoma differentiation-associated protein 5) and RIG-1 (retinoic acid-inducible gene I) results in activation of TRIF, followed by activation of TANK-binding kinase 1 (TBK1), an enzyme that catalyses the phosphorylation of IRF-3 (interferon regulatory factor 3). Phosphorylated IRF-3 then forms dimers, which translocate to the nucleus and activate transcription of type I interferon genes (24, 25). Type I IFN production can be amplified by a positive feedback loop (26). Secreted IFNs bind and activate the type I IFN receptor IFNAR (interferon-alpha/beta IFN-receptor) leading to Stat1 (signal transducer and activator of transcription) and Stat2 phosphorylation *via* JAK protein kinase JAK1 and Tyk2. After dimerization Stat1 and Stat2 together with IRF9 forms the transcription factor complex ISGF3 (interferon-stimulated gene factor 3) which by binding to ISRE (IFN-stimulated response elements) induces expression of IFN-inducible genes (ISGs) (27, 28). Double stranded RV RNA can also enter endocytotic compartments where it is potentially recognized by TLR3. Upon ligand recognition TLR3 can recruit TRIF (TIR-domain-containing adapter-inducing interferon-β) and TRAM (TRIF- related adaptor molecule) (29) which signal for IRF3 activation and IFN-β production. IRF3 becomes phosphorylated by TBK1 or IKK1 (IκB kinase 1) (24, 30, 31). After binding of LPS to the TLR4 complex, signalling occurs *via* two pathways: the MyD88 (myeloid differentiation factor 88) -TIRAP (TIR domain containing adaptor protein) and the TRAM-TRIF pathway (24). The MyD88-TIRAP pathway which is mainly but not entirely responsible for induction of pro-inflammatory cytokines involves activation of NFκB and AP1 and MAPKs such as extracellular signal-regulated kinase (ERK) and c-Jun N-terminal kinase (JNK) (29) while TRAM and TRIF after internalization of the LPS-TLR4 complex signal to TBK1 for IRF3 activation and primary IFN production (29).

Having shown that exposure to LPS following infection with rubella alters the biological response of GM-MΦ and M-MΦ we tested whether distinct components of the virus and LPS-dependent signalling pathways are affected by the treatment. The role played by components in these pathways is outlined in a short and simplified way in **Figure 4**.

**Figure 5:**
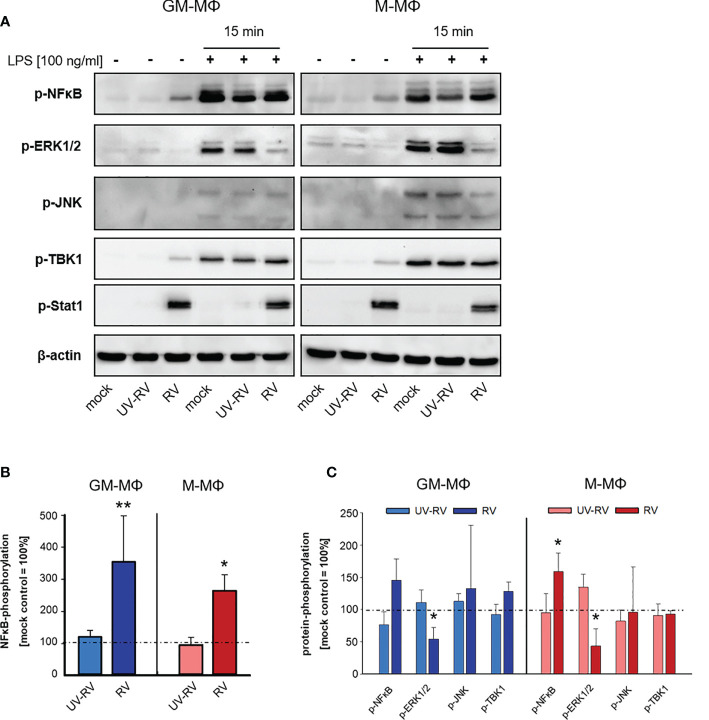
LPS-induced signal transduction after infection with RV. **(A)** 24 h after incubation RV-, UV-RV- and mock-infected MΦ (5x10^5^/ml) were incubated in the presence and absence of LPS (100 ng/ml). After 15 min cells were lysed using RIPA buffer and protein fractions were separated by SDS-PAGE and subjected to western blot analysis using Ab specific for the indicated proteins. One representative blot out of three is shown. Western blot bands of mock-, UV-RV- and RV-infected cells incubated without **(B)** and with LPS (100 ng/ml) **(C)** were quantified as relative intensities after normalization to the loading control β-actin. Data are presented as percent of optical density of the respective mock control (= 100%) and as means ± SD (n = 3). Statistical analysis was performed using the ANOVA test and significances were calculated to the mock control. *p ≤ 0.05, **p ≤ 0.01.

As shown in **Figures 5A, B** we found the transcription factor NFκB to be phosphorylated in response to RV infection. NFκB plays an important role in the gene-regulatory network in immune responses including the induction of the genes that encode IFN-β and pro-inflammatory cytokines (32, 33). It associates with the transcription factors AP1 and IRF3 resulting in the direct binding and activation of the IFN-β promoter (34).

After RV-infection we detected only minor amounts of phosphorylated TBK1 (**Figure 5A**), a central kinase in the pathway leading to type I IFN-production. However, p-Stat1 expression was greatly enhanced in both MΦ subtypes, which was consistent with an increased production of IFNs following infection with RV.

Next, we tested the effect of LPS on the phosphorylation profile of NFκB, ERK1/2, JNK, TBK1 and Stat1 when applied 24 h after virus infection.

As shown in **Figures 5A, C** treatment of controls (mock, UV-RV) with LPS had no effect on Stat1 phosphorylation and led to an increased phosphorylation of NFκB, ERK1/2, JNK and TBK1, indicating that both TLR4 dependent pathways have been engaged.

Prior infection with RV resulted in an increased phosphorylation of NFκB (M-MΦ), while it caused a drastic downregulation of p-ERK1/2 in both subtypes. This effect was also slightly visible in the absence of LPS challenge.

In conclusion, an established RV infection leads to a decrease of the LPS-induced ERK1/2 phosphorylation in both MΦ subtypes.

### RV Dampens LPS-Induced Glycolysis

Having shown that exposure of GM- and M-MΦ to RV prior to incubation with LPS results in an enhanced production of IFNs, we asked whether activation by the respective stimuli alone or applied successively is associated with metabolic changes.

To determine the metabolism of GM- and M-MΦ after RV-infection followed by exposure to LPS we measured extracellular acidification rate (ECAR) as an index of lactic acid production and oxygen consumption rate (OCR) as an index of oxidative phosphorylation.

**Figure 6:**
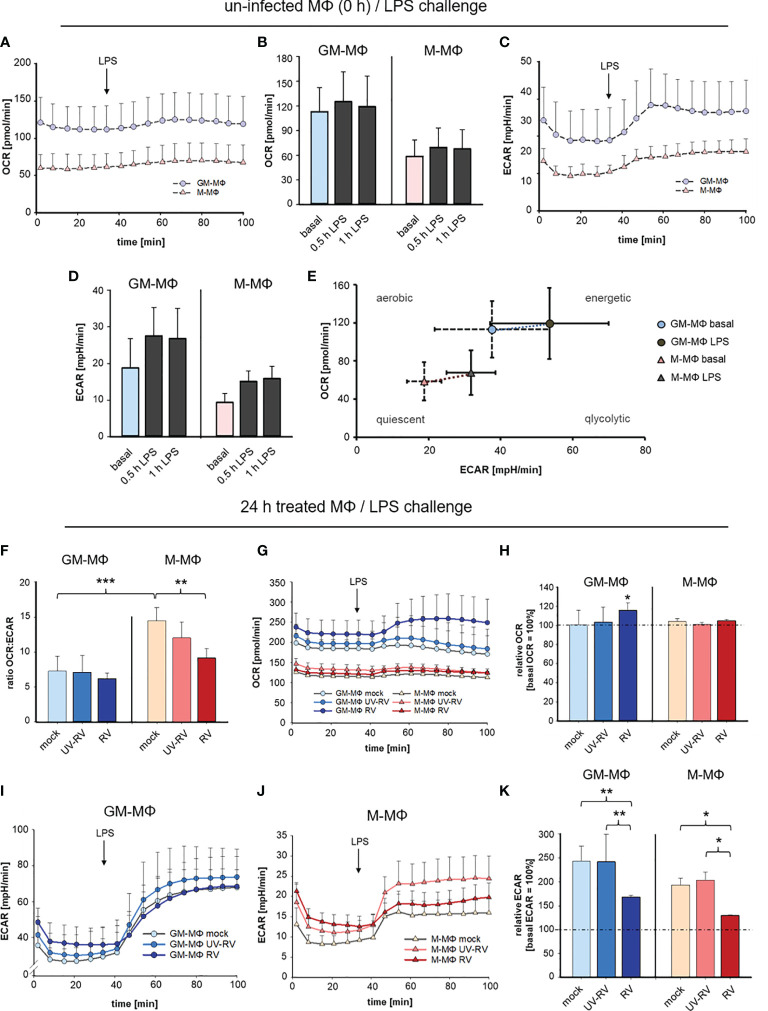
LPS-induced metabolic response after infection with RV. Extracellular flux analysis was performed in an XFp analyser and metabolic activity in MΦ was measured as oxygen consumption rate (OCR) and extracellular acidification rate (ECAR). OCR and ECAR were measured at 16 measurement points every 6 min. Graphical representation of OCR **(A, B)** and ECAR **(C, D)** of GM-MΦ and M-MΦ treated with LPS (100 ng/ml). Data represent means ± SD (n = 3). Detailed analysis of OCR **(B)** and ECAR **(D)** at 0.5 h and 1 h after exposure to LPS. **(E)** Energy Phenotype Profiles of basal and 0.5 h LPS-stimulated GM-MΦ and M-MΦ are shown. Data represent means ± SD (n = 3). **(F–K)** At 24 hpi mock-, UV-RV- and RV-infected GM- and M-MΦ were measured in extracellular flux analysis. **(F)** The ratio of the OCR to ECAR (OCR_basal_/ECAR_basal_) at basal conditions (before application of LPS) was calculated as means ± SD (n = 3). Statistical analysis was performed using the ANOVA test. Graphical representation of OCR **(G)** and ECAR **(I, J)** of MΦ (24 hpi) exposed to LPS (100 ng/ml). **(H, K)** Values represent the percentage change of OCR and ECAR 30 min after exposure to LPS relative to basal measurements (= 100%). Statistical analysis for **(H, K)** was performed using the ANOVA test and significances were calculated to basal OCR or ECAR (= 100%). *p ≤ 0.05, **p ≤ 0.01, ***p ≤ 0.001.

OCR and ECAR were measured every 6 min at 16 measurement points. As seen in **Figures 6A–D** GM-MΦ exhibited elevated basal OCR and ECAR levels compared to M-MΦ indicating that the bioenergetics profile differs between the twoMΦ subsets. Challenge with LPS for 0.5 h and 1 h induced a profound increase in ECAR in GM-MΦ and to a lesser extent in M-MΦ, whereas OCR remained unaffected (**Figures 6B, D**).

These data as illustrated in the Energy Phenotype Profile of ECAR and OCR (**Figure 6E**) demonstrate that M-MΦ are in a more quiescent state than GM-MΦ. In response to LPS GM-MΦ undergo metabolic reprogramming towards glycolysis slightly more than M-MΦ.

As a next step, the bioenergetics profile that is reflected by the ratio of OCR and ECAR was determined in MΦ having been infected with RV and UV-RV for 24 h (**Figure 6F**).

The OCR/ECAR ratio serves as a useful parameter to compare metabolic states between different cell types (35). The lower the ratio, the higher the glycolytic activity.

The ratio analysis of GM- and M-MΦ 24 hpi (**Figure 6F**) confirmed their metabolic identity, which relies on glycolysis and oxidative phosphorylation, respectively.

Notably in M-MΦ, this ratio is lower after infection with RV than in the mock-control (**Figure 6F**) suggesting that during RV-infection glycolytic activity is increased in M-MΦ.

When determining the metabolic activity after LPS-challenge OCR values slightly increased in RV- and to a lesser extent in UV-RV- and mock-infected GM-MΦ whereas M-MΦ did not respond to LPS (**Figure 6G**).

Compared to basal OCR levels the rise at 0.5 h after treatment with LPS was only significant in RV-infected GM-MΦ and at 24 hpi (**Figure 6H**).

Independent of the preceding treatment both MΦ subtypes responded to LPS with a substantial increase in ECAR levels (**Figures 6I, J**). In relation to basal ECAR the values reached 0.5 h after LPS treatment were significantly lower in RV-infected MΦ than in mock and UV-RV treated controls (**Figure 6K**).

Taken together these data indicate that stimulation with LPS resulted in a slight increase in glycolytic activity of both GM- and M-MΦ. During RV-infection glycolytic activity was slightly increased in M-MΦ, while in both cell types RV-infection significantly dampened the LPS-induced increase in glycolysis. This latter metabolic reprogramming indicates that RV signalling followed by LPS signalling results in a specific biological response, which is associated with a diminished glycolysis.

**Figure 7:**
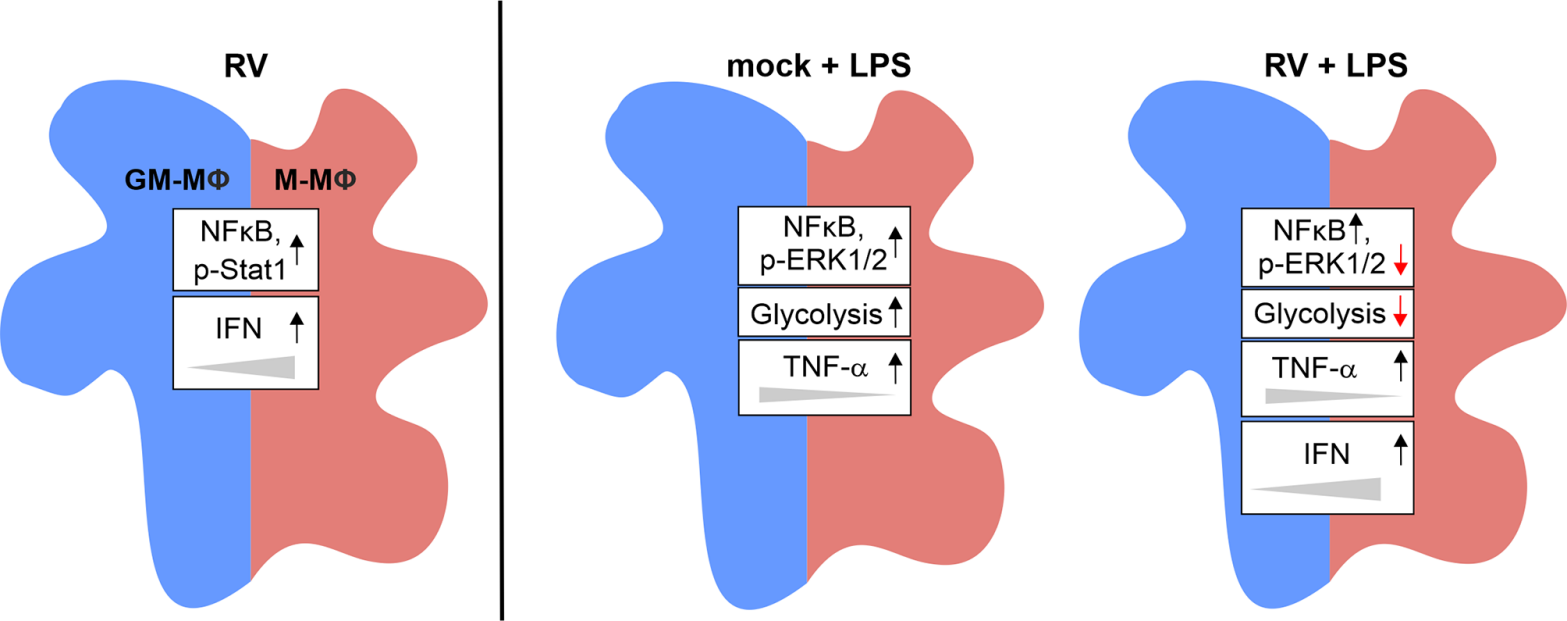
Graphical summary of the data presented. Exposure of GM- and M-MΦ to RV for 24 h results in altered metabolic and signal transduction pathways and in increased IFN levels preferentially in M-MΦ. Co-stimulation with LPS revealed differential responses in metabolic events, signal transduction mechanisms and cytokine response between RV and mock infection.

As an overall conclusion, the here presented data are summarized in **Figure 7**.

## Discussion

MΦ as central components of the innate and adaptive immune response are well known for their antiviral functions (11). To what extent they contribute to RV pathogenesis and to the outcome of a viral response when being infected themselves is less well understood.

Here we show in line with previous reports (11) that RV infects GM- and M-MΦ and replicates within these cells. As described for other virus-related processes we show that productive virus replication which is missing after UV-induced RV inactivation is required to trigger TNF-α and IFN-production in GM- and M-MΦ (1). Consistent with the cytokine profile of the two MΦ populations, M-MΦ produced far more IFNs than GM-MΦ in response to RV, confirming a more anti-viral response of M-MΦ.

However, the TNF-α response was low compared to the amounts produced after exposure to LPS and differences between the two subtypes were hardly visible. Thus, the induction of this cytokine during RV infection does not represent a major component of the defense mechanism of RV-infected MΦ.

Of the three IFNs tested, production of IFN-λ1 (type III IFN) was found to be highest. A similar IFN-response profile was detected after infection of human alveolar basal epithelial A549 cells with RV (11). Type III IFNs exhibit several common features with type I IFNs (IFN-α/β) (36). They appear to be induced by the same stimuli and similar to IFN-α/β they use the JAK/Stat signalling pathway. In contrast to IFN-α/β which binds to IFNAR, a receptor composed of the IFNAR1 and IFNAR2 chains (37, 38), IFN-λ1 uses a receptor (IFNLR1) consisting of the IFNLR1 and IL-10R2 chain (39, 40). As the RV-induced production of IFNs can be amplified by a positive feedback loop through binding to the respective receptors as it was described for other virus infections (27, 28) we reasoned that expression levels of the receptor may contribute to the differences in the cytokine response by GM- and M-MΦ. We found that IFNLR1 and IFNAR1 transcripts to be expressed at a similar level in both subtypes. However, the expression levels not only increased in RV-infected cells during cultivation, but also in controls, indicating that the abundance of the IFNAR1 and IFNLR1 transcripts was not related to the IFN-response.

Next, we extended our studies by exposing RV-infected MΦ to a second stimulus to mimic co-infections and to characterize their inflammatory and metabolic status in association with a viral infection. We chose RV as a primary pathogen as RV replicates in both types of human MΦ and the bacterial component lipopolysaccharide (LPS) as a secondary stimulus and determined the response to the combined treatment. LPS, the major constituent of the outer membrane of all gram-negative bacteria, is a potent activator of MΦ responses involved in the host defense against infections (41). Due to its well-characterized effects on MΦ including the induction of the potent pro-inflammatory cytokine TNF-α, this intensively studied compound is best suited to examine the effects of bacterial infections on MΦ. Exposure of MΦ to RV followed by washing 24 hours post-infection and subsequent incubation for 6 h resulted in a low IFN-response compared to amounts reached after virus infection at 24 hpi. This decline of the antiviral IFN-response over time of infection could contribute to the still unclear mode of persistence of reactivated RV vaccine strain in M2 MΦ in PID patients (10). Moreover, the replication of RV in MΦ could support virus dissemination within the body, as RV was successfully isolated from mononuclear cells from synovial fluid as well as from peripheral blood (42). Such a mode of MΦ-assisted viral dissemination was described for several viruses including the cell-to-cell based transfer of measles virus from infected MΦ to epithelial cells (3, 43).

Although the IFN-β and -λ1 protein concentrations drastically declined under these assay conditions, the mRNA levels remained elevated. Consistent with this finding, *in vitro* transcription/translation reactions have shown, that viral capsid protein downregulates protein synthesis. The capsid protein interacts with poly(A) binding protein (PABP), which seems to interrupt the binding of PABP to the translation initiation complex (IF4F) (44).

Translational and transcriptional activity started again when LPS was added, indicating that RV primed the cells to react to the LPS-induced stimuli. A similar response was obtained when RV was substituted by IFN-β supporting the presumption that IFN-β might be an important priming stimulus induced by RV. Either RV infection or low amounts of exogenously added IFN-β (20 pg/ml) shown to be sufficient to induce phosphorylation of Stat1 a transcription factor important for production of ISGs. This indicates a role of IFN-β in priming of MΦ for enhanced production of IFNs in response to other stimuli.

It has been sheen that priming mechanisms seem to be of great importance (27), when the amounts of IFNs are limiting and augmentation of IFN-signalling is required to meet viral and bacterial challenges (45, 46). At present it is unclear to what extent the presence of the virus within the two MΦ subsets contributes to the LPS-induced renewed increase in IFN-signalling.

The here used stimulus LPS added 24 h after incubation in the absence of RV does not induce IFN-production. It initiates TRIF-dependent phosphorylation of TBK1, which would be consistent with an enhanced IFN-production but the missing phosphorylation of Stat1 does not support this assumption. The full activation of the TRIF-TBK1-IRF3/7-Stat1 pathway and augmentation of IFN-production requires RV.

To our surprise we found ERK1/2, a member of the MAP-kinase (MAPK) family, to be downregulated after pre-infection with RV. ERK1/2 which becomes activated not only by engagement of LPS signalling (47) as shown here but also in response to type I IFNs (45, 48), phosphorylates and thus activates Stat1. Stat1 in turn enhances either independently or together with NFκB and AP-1 transcriptional activity and downstream ISG (interferon-stimulated gene) induction. As this enhancement is dependent on the activation of the kinases it is difficult to envisage how inactivation of ERK1/2 as described here, contributes to the noted increase in cytokine production especially after LPS challenge. For the RK13 kidney cell line, it was shown that RV infection led to increasing levels of phosphorylated ERK and that inhibition of Ras-Raf-MEK-ERK signalling resulted in reduced RV replication (49).

Unlike the here described RV-induced priming effect, infection of MΦ with human rhinovirus instead of RV has been reported to impair the cytokine response to LPS (50). Thus, the type of virus seems to be crucial for the outcome of a secondary bacterial stimulation and no predictions can be made.

In this study we also provide evidence that activation of GM- and M-MΦ with either bacterial or viral stimuli not only results in functional but also in metabolic changes. Consistent with previous reports we found that exposure to LPS caused an increase in glycolysis (51, 52) a metabolic shift proven to be critical not only for enhanced production of pro-inflammatory cytokines, notably IL-1β (53) but also for other monocytic functions, such as processes involved in leucocyte adhesion cascades (54).

However, less well studied is the role of glycolysis in mediating macrophage/monocyte activation and function following viral infection. It has been shown that in MΦ cytosolic viral recognition by way of secondary IFN signalling results in upregulation of glycolysis in a PFKFB3 (6-phosphofructo-2-kinase/fructose-2,6-biphosphatase 3) dependent manner (55). Engagement of this pathway supported the engulfment and removal of virus-infected cells thus providing evidence that glycolysis is closely related to anti-viral activity in these immune cells (55). RV infection also resulted in a slight upregulation of glycolysis preferentially in M-MΦ, however, it dampened glycolysis induced by challenge with LPS in both MΦ subsets. The contribution of the RV-induced IFN to the observed decrease in glycolysis needs to be addressed in further studies. Recently it was shown for murine bone marrow-derived MΦ that the application of IFN-β restrains glycolysis (56).

Surprisingly despite a reduced glycolytic activity oxidative phosphorylation was not affected, indicating that glycolysis was not coupled to cellular respiration. Similar observations have been published previously (48) but inhibition of oxidative phosphorylation coupled with a corresponding increase in glycolysis has also been described (20). This metabolic reprogramming seems to be of great importance as the cells are faced with different functional demands.

In summary by investigating not only metabolic but also functional and signalling events in MΦ exposed to viral infection and bacterial stimulation in succession we here contribute to a field of great clinical importance. Our data add to the understanding of the still ill-defined involvement of MΦ in prenatal and postnatal RV infection. Moreover, these immune cells could also be involved in the still undercharacterized mode of vertical transmission of RV during pregnancy. In the case of Zika virus transmission during pregnancy placental MΦ were shown to be permissive to Zika virus and maternal blood is the likely source of the infection of the placenta (57, 58). We found that human MΦ infected with RV to become more responsive to a bacterial stimulus, thus amplifying the cytokine response and inducing changes in metabolic reprogramming and in signal transduction mechanisms.

## Data Availability Statement

The raw data supporting the conclusions of this article will be made available by the authors, without undue reservation.

## Ethics Statement

The studies involving human participants were reviewed and approved by Medical Faculty, Leipzig University, Liebigstraße 18, 04103 Leipzig. The patients/participants provided their written informed consent to participate in this study.

## Author Contributions

SH, CC, AG, and ES contributed to conceptualisation. ES, AG, CC, and LP carried out the experiments. ES, SH, and CC wrote the first draft of the manuscript. SH, CC, AG, and UK reviewed and edited the draft of the manuscript. All authors contributed to final manuscript revision, read and approved the submitted version.

## Conflict of Interest

The authors declare that the research was conducted in the absence of any commercial or financial relationships that could be construed as a potential conflict of interest.

## Publisher’s Note

All claims expressed in this article are solely those of the authors and do not necessarily represent those of their affiliated organizations, or those of the publisher, the editors and the reviewers. Any product that may be evaluated in this article, or claim that may be made by its manufacturer, is not guaranteed or endorsed by the publisher.
